# Green-chemistry Compatible Approach to TiO_2_-supported PdAu Bimetallic Nanoparticles for Solvent-free 1-Phenylethanol Oxidation under Mild Conditions

**DOI:** 10.1007/s40820-015-0044-6

**Published:** 2015-06-09

**Authors:** Jian-Bing Chang, Chang-Hai Liu, Jie Liu, Yu-Yan Zhou, Xu Gao, Sui-Dong Wang

**Affiliations:** Soochow University-Western University Joint Centre for Synchrotron Radiation Research, Jiangsu Key Laboratory for Carbon-Based Functional Materials & Devices, Institute of Functional Nano & Soft Materials (FUNSOM), Soochow University, Suzhou, 215123 Jiangsu People’s Republic of China

**Keywords:** PdAu nanoparticles, TiO_2_ nanosupport, Electronic structure

## Abstract

TiO_2_-supported PdAu bimetallic nanoparticles (NPs) with small size and good dispersity were prepared by the room-temperature ionic liquid-assisted bimetal sputtering, which is simple, environmentally friendly, and free of additives and byproducts. Pd/Au atomic ratio can be tuned by controlling the sputtering conditions simply. High catalytic activity was found in PdAu–NPs–TiO_2_ hybrids for solvent-free selective oxidation of 1-phenylethanol using O_2_ as the oxidant at the low temperature of 50 °C and low pressure of 1 atm. It was found that Pd/Au ratio strongly affected the catalytical activity, and the highest conversion of about 35 % and turnover frequency of about 421 h^−1^ were achieved at 1:1 of Pd/Au atomic ratio. The synergistic effect in PdAu NPs was also discussed based on the comprehensive characterization results. The present approach may offer an alternative platform for future development of green-chemistry compatible bimetallic nanocatalysts.

## Introduction

The selective oxidation of primary alcohols to aldehydes/ketones is crucially important in both laboratory and commercial processes [1–4]. In homogeneous catalysis for conversion of alcohols to aldehydes, it is often needed to use organic solvents, which are typically expensive, toxic, and harmful to the environment [5]. Thus, great efforts have been made to design and synthesize alternative catalysts which can use molecular O_2_ as the oxidant. However, the use of O_2_ under severe conditions such as high temperature and high pressure could induce issues of overoxidation, decomposition products, and/or explosion risk. It is hence highly desired to realize the efficient catalysis free of solvents under mild conditions. Toward this target, metal nanoparticles (NPs) such as Au NPs [6–15], Pd NPs [16, 17] and PdAu bimetallic NPs [18–24] have been demonstrated to be promising catalysts for solvent-free selective oxidation of alcohols [8, 10, 11, 20, 21]. Especially, PdAu bimetallic NPs often exhibit superior catalytic performance than their monometallic counterparts due to the synergistic effects [18–24]. In order to maintain the small size and good dispersion of PdAu NPs, which are beneficial for the catalysis, nanosupports have been extensively utilized to stabilize the bimetallic NPs on top.

The conventional way to synthesize supported metal NPs involves reduction of corresponding metal salts, where additives such as capping agents and byproducts are always present [25–28]. In some cases, high-temperature processing is required as well [29, 30]. Recently, we have developed a more environmentally friendly strategy using the room-temperature ionic liquid (RTIL)-assisted sputtering to prepare supported metal NPs [31, 32], which is simple, clean, and totally free of additives and byproducts. By the green-chemistry compatible method, PdAu NPs are successfully decorated on TiO_2_ NPs which act as the nanosupport. The comprehensive results demonstrate the bimetallic nature of the PdAu NPs, and charge redistribution is observed among Pd, Au, and TiO_2_. The TiO_2_-supported bimetallic NPs show good catalytic capability for the selective oxidation reaction of 1-phenylethanol to acetophenone, which can occur near room temperature without a solvent. Furthermore, the catalytic activity of the PdAu NPs is significantly dependent on Pd/Au ratio, suggesting a strong correlation among the catalytic performance, bimetallic composition, and charge redistribution.

## Experimental

### Materials

Pd and Au targets for the sputtering were purchased from Hefei Kejing. 1-phenylethanol was purchased from Sinopharm Chemical Reagent. Pristine TiO_2_ NPs with an average diameter of 25 nm were acquired from Hangzhou Wanjing. The RTIL, 1-Butyl-3-methylimidazolium tetrafluoroborate ([BMIm][BF_4_], purity >99 %), was purchased from Shanghai Chengjie Chemical, which was dried in vacuum for 24 h before use.

### Synthesis of TiO_2_-supported PdAu NPs

**Scheme 1:**
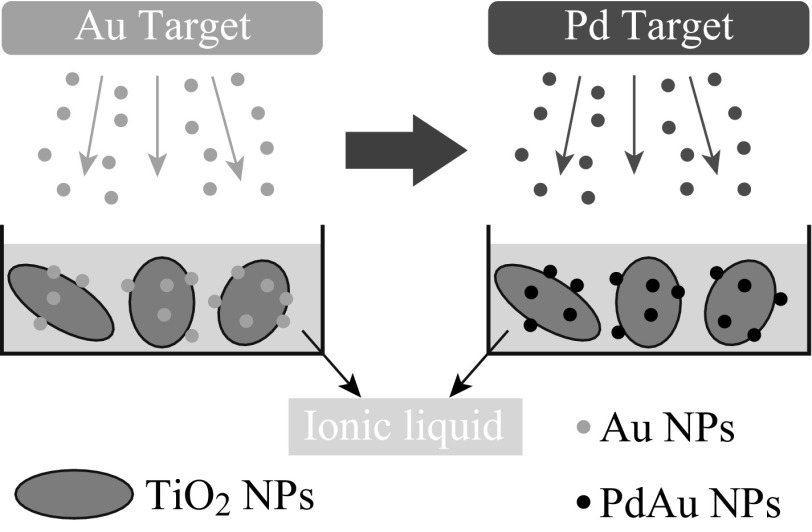
Preparation method for PdAu–NPs–TiO_2_ hybrids: Successive sputtering of Au and Pd onto RTIL of [BMIm][BF_4_], in which TiO_2_ NPs are pre-dispersed

As depicted in Scheme 1, the TiO_2_-supported PdAu NPs were prepared by successive sputtering of Au and Pd onto the TiO_2_–RTIL suspension [31, 32]. Firstly, 10 mg TiO_2_ NPs were adequately dispersed into 10 mL [BMIm][BF_4_] with ultrasonication. Secondly, the TiO_2_–RTIL suspension was dropped into a clean stainless steel pot, and then Au was sputtered onto the suspension at room temperature in a desktop sputtering system (Quorum Technologies, equipped with a thickness monitor which is pre-calibrated by a surface profiler). Thirdly, Pd was sputtered onto the Au–TiO_2_–RTIL suspension by simply altering the metal target from Au to Pd. During the sputtering, Ar working pressure and deposition rate were kept at about 0.01 mbar and 0.2 Å s^−1^, respectively. The total sputtering time was controlled to be same for all the samples to get a same metal loading, e.g., 40 s for Au plus 40 s for Pd to the PdAu bimetallic NPs, and correspondingly 80 s for Au/Pd to the monometallic NPs. Eventually, the metal–NP–TiO_2_ hybrids were separated from [BMIm][BF_4_] by repeated high-speed centrifugation and decantation. Note that the current approach is a general method, which can be used to prepare various bimetallic NPs on diverse nanosupports [31, 32].

### Characterization of TiO_2_-supported PdAu NPs

The crystalline structures of the metal–NPs–TiO_2_ hybrids were characterized with X-ray diffraction (XRD, PANalytical Empyrean) with Cu *Kα* radiation. The microscopic structures of the metal–NPs–TiO_2_ hybrids were investigated with high-resolution transmission electron microscopy (HRTEM, FEI Quanta FRG 200F, operating at 200 kV) and high-angle annular dark-field scanning transmission electron microscopy (HAADF-STEM). The UV–Vis absorption measurements were carried out on a UV–Vis–NIR spectrophotometer (PerkinElmer, Lambda 750). The electronic structures of the metal–NPs–TiO_2_ hybrids were characterized with X-ray photo-emission spectroscopy (XPS, Kratos Axis Ultra DLD, monochromatic Al *Kα*). The X-ray absorption near-edge structure (XANES) measurements were performed at the BL14W1 beamline of the Shanghai Synchrotron Radiation Facility (SSRF).

### Catalytic Reaction with TiO_2_-supported PdAu NPs

The solvent-free oxidation reaction of 1-phenylethanol using O_2_ was conducted in a glass flask pre-filled with 5 mL 1-phenylethanol and 8 mg hybrid catalyst. The mixture was stirred and heated using a magnetic stirrer with heating function. The system was equipped with a thermocouple to control the temperature and a reflux condenser to recover the vaporized solution. In each reaction run, the mixture was raised to 50 °C under stirring (1000 rpm). O_2_ was bubbled into the mixture at a constant flow rate (250 mL min^−1^) to initiate the reaction. After the reaction lasting 5 h, the reaction products were quantitatively analyzed with gas chromatograph (GC, Agilent).

The conversion, selectivity, and turnover frequency (TOF) are defined as follows:

$$ {\text{Conversion }}\left( \% \right) \, = \,\,\frac{{\text{mol of reactant converted}}}{{\text{mol of reactant in feed }}} \, \times { 1}00 \, \% $$

$$ {\text{Selectivity }}\left( \% \right) \, = \frac{{\text{mol of target formed}}}{{\text{mol of reactant converted }}} \, \times { 1}00 \, \% $$

$$ {\text{TOF }}\left( {{\text{h}}^{ - 1} } \right) \, = \frac{\text{mol of reactant converted}}{{ ( {\text{mol of total metal)}}\,D_{\text{m}} t \, }} \, $$

where the amount of reactant and target product are measured by GC; the amount of total metal are approximated as the amount of Au and Pd atoms, which are measured by inductively coupled plasma atomic emission spectroscopy (ICP-AES); *D*_m_ is the dispersivity of metal; and *t* is the reaction time.

## Results and Discussion

### Microscopic Structure

**Fig. 1:**
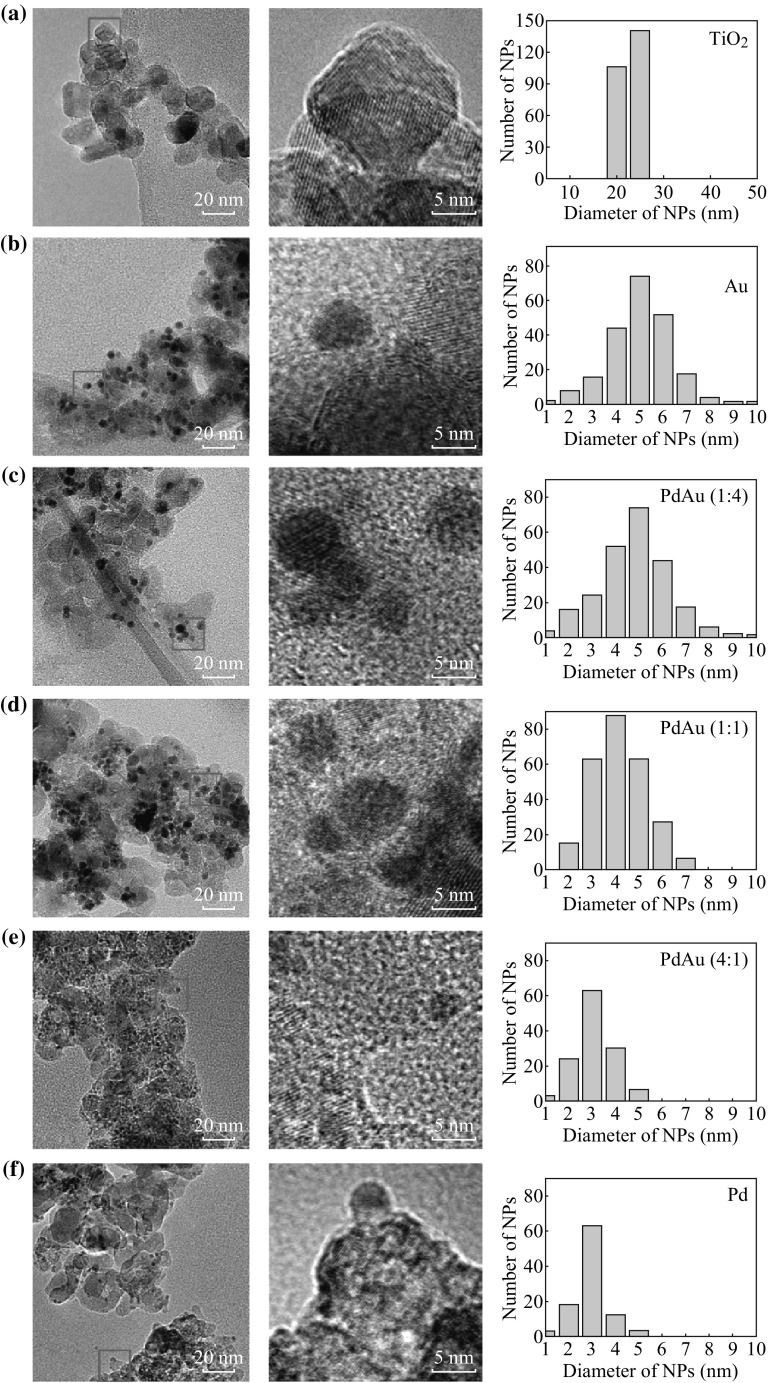
TEM images and NPs size distributions of **a** TiO_2_ NPs as the support, **b** Au NPs on TiO_2_, **c** PdAu (1:4) NPs on TiO_2_, **d** PdAu (1:1) NPs on TiO_2_, **e** PdAu (4:1) NPs on TiO_2_, and **f** Pd NPs on TiO_2_. TEM images in the *middle* are magnified* square region* shown in the *left*

Commercially available TiO_2_ NPs with an average diameter of 25 nm were used as the nanosupport, and the morphology is shown in Fig. 1a. Utilizing the RTIL-assisted sputtering method, Au/Pd monometallic NPs or PdAu bimetallic NPs were decorated on the TiO_2_ support, where Pd/Au ratio (atomic ratio, unless otherwise noted) was controlled by adjusting the sputtering conditions of Au and Pd [32]. It was noted that the TiO_2_ support played a role of stabilizing metal NPs on top, otherwise the metal NPs will aggregate in cleaning process for removing [BMIm][BF_4_] if the support is absent [31]. Figure 1b–f displays the TEM images and metal nanoparticle size distributions of the Au–TiO_2_, PdAu (1:4)–TiO_2_, PdAu (1:1)–TiO_2_, PdAu (4:1)–TiO_2_, and Pd–TiO_2_ hybrids, respectively. It can be seen that the size of Au monometallic NPs is larger than that of Pd monometallic NPs, and the size of PdAu bimetallic NPs decreases gradually from about 5 to 3 nm upon increasing Pd/Au ratio.

**Fig. 2:**
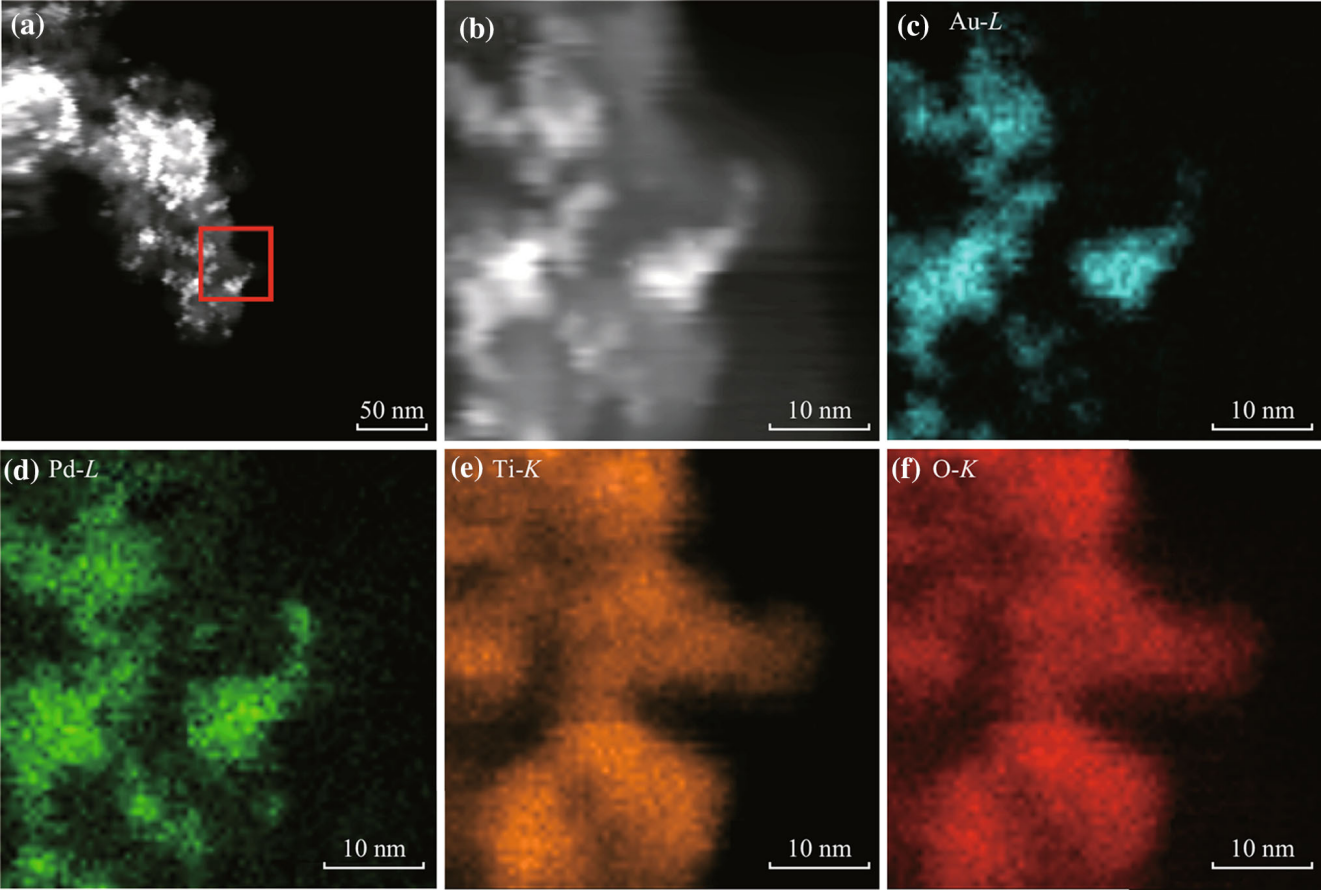
HAADF-STEM images of PdAu (1:1) NPs on TiO_2_ **a** in *large scale* and **b** in selective *small scale*. Elemental mapping for **c** Au, **d** Pd, **e** Ti, and **f** O

In order to clarify the microscopic structure of PdAu NPs, the elemental spatial distribution of Au and Pd is probed by mapping of the energy-dispersive spectroscopy (EDS). Figure 2a–d illustrates the HAADF-STEM images for the sample of PdAu (1:1)–TiO_2_, where Au and Pd spatial distributions are almost overlapped with each other. Similar HAADF-STEM results were observed for the samples with other Pd/Au ratios, and it suggests that Au and Pd are intermixed in the PdAu NPs due to atomic inter-diffusion [33–37]. The strong intermixing of Au and Pd can provide a large number of Au/Pd interfaces, which may be in favor of promoting catalytic activity of PdAu NPs [38]. On the other hand, the Ti and O spatial distributions are consistently uniform, as demonstrated in Fig. 2e, f.

**Fig. 3:**
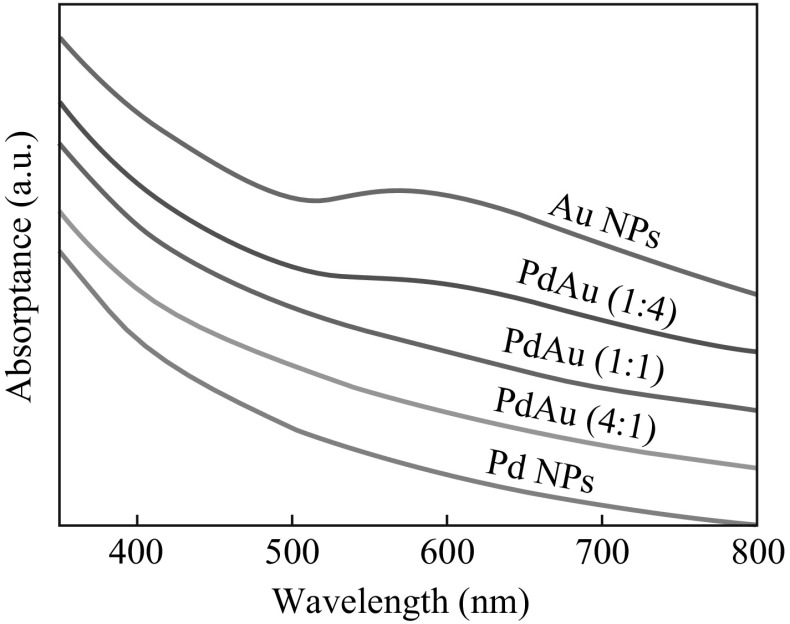
UV–Vis absorption spectra of TiO_2_-supported metal NPs with different Pd/Au atomic ratios

Figure 3 shows the UV–Vis absorption spectra of the TiO_2_-supported metal NPs with different Pd/Au ratios, in which the surface plasmon resonance (SPR) band of Au is around 570 nm. There is a small red-shift compared with the SPR band of typical Au NPs (~530 nm), which could be caused by the electronic structure modification of Au NPs due to the electron transfer from TiO_2_ to Au [39–43]. Upon addition of Pd, the SPR band gradually disappears. The disappearance of the SPR feature supports the HAADF-STEM-derived claim that Au and Pd are intermixed in the PdAu NPs. Therefore, the PdAu NPs are concluded to be bimetallic NPs rather than a physical mixture of separate Au and Pd monometallic NPs.

### Electronic Structure

**Fig. 4:**
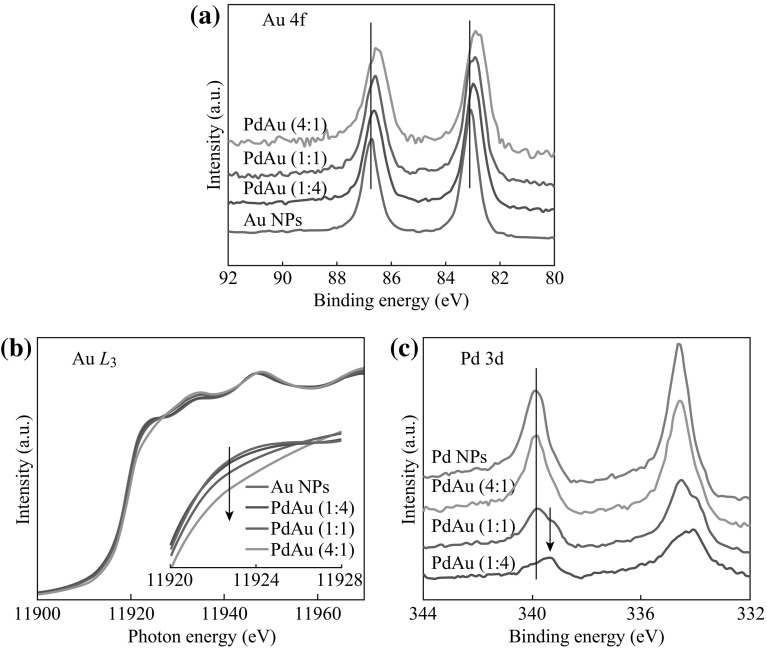
**a** XPS Au 4*f* spectra, **b** XANES Au *L*_3_-edge spectra, and **c** XPS Pd 3*d* spectra of TiO_2_-supported metal NPs with different Pd/Au atomic ratios. *Inset* in **b** is magnified *whiteline region*, where Au *L*_3_-edge jump is normalized

The charge redistribution in PdAu NPs is another bimetallic signature, which is investigated by XPS and XANES based on synchrotron radiation. Figure 4a shows the XPS Au 4*f* spectra, where the binding energy of Au 4*f*_7/2_ peak for Au monometallic NPs is at about 83.0 eV. Considering the binding energy of bulk Au 4*f*_7/2_ peak (typically at about 84.0 eV), there is a significant negative shift in binding energy (corresponding to electron gaining) for the Au NPs on TiO_2_. As TiO_2_ NPs often show *n*-type behavior arising from the oxygen vacancies, electron transfer from the TiO_2_ support to the Au NPs may be responsible for the binding energy shift [44]. Upon addition of Pd, the Au 4*f* peaks are further negatively shifted (up to 0.3 eV) with increasing Pd/Au ratio. It implies a strong interaction between Au and Pd [39, 44, 45], and electron transfer from Pd to Au may occur and result in a negative shift in binding energy. Furthermore, when Pd/Au ratio is increased, the full width at half maximum (FWHM) of the Au 4*f* peaks is broadened. This can be attributed to the size reduction of PdAu NPs with increasing Pd/Au ratio, as shown in Fig. 1.

Figure 4b shows the XANES Au *L*_3_-edge spectra of the TiO_2_-supported metal NPs with different Pd/Au ratios, where the absorption jump (whiteline) region around 11,925 eV is magnified as the inset. The whiteline characterizes the dipole transition from Au 2*p* to 5*d* orbitals, and thus its intensity corresponds to the number of Au *d*-band holes. Compared with Au monometallic NPs, the whiteline intensity is decreased for PdAu bimetallic NPs, and the sample with higher Pd/Au ratio shows lower whiteline intensity. The trend of the whiteline change may be due to electron transfer from Pd to Au, which is in accordance with the XPS Au 4*f* results and literature [46].

On the other hand, the XPS Pd 3*d* spectra of the TiO_2_-supported metal NPs with different Pd/Au ratios are shown in Fig. 4c. The binding energy of Pd 3*d*_3/2_ peak for Pd monometallic NPs is at about 339.8 eV, which is lower than that of bulk Pd at about 340.4 eV. Similar to the Au NPs on TiO_2_, the negative shift in binding energy can be ascribed to electron transfer from the *n*-type TiO_2_ support to the Pd NPs. Upon addition and promotion of Au, the original Pd feature is gradually weakened, and a new Pd feature at lower binding energy emerges and grows up. This could be a result of strong interaction between Au and Pd as observed in PdAu alloys [33, 45]. According to the above, the charge redistribution is present in the PdAu NPs, plausibly Pd losing *s*, *p*-electrons and getting *d*-electrons with net electron loss, while Au gaining net *d*-electrons [33, 43, 44, 46, 47].

### Crystalline Structure

**Fig. 5:**
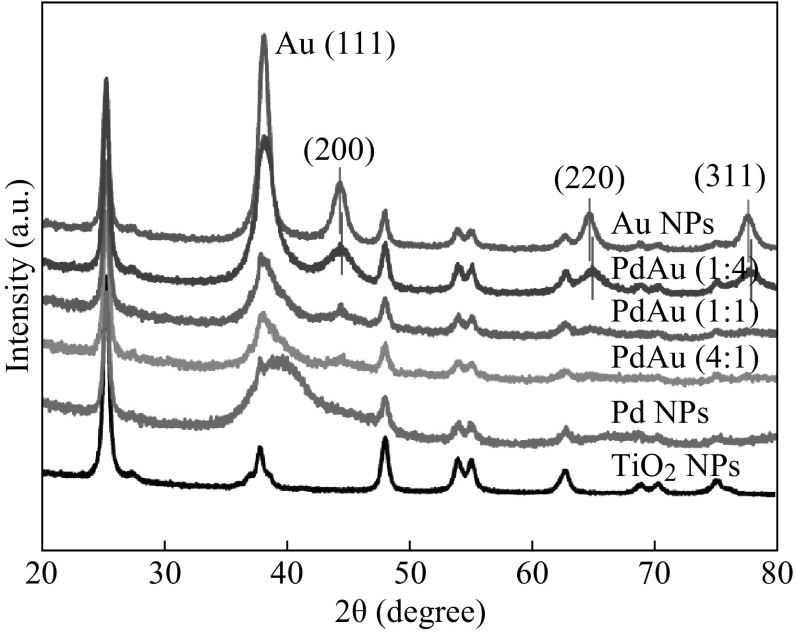
XRD patterns of TiO_2_ NPs (anatase) and TiO_2_-supported metal NPs with different Pd/Au atomic ratios

The XRD patterns of the metal–NPs–TiO_2_ hybrids are shown in Fig. 5. The TiO_2_ support shows the crystalline features of anatase phase, which remains identical 2*θ* positions in the hybrids. Thus, there is no phase change or crystallinity degradation of TiO_2_ after the metal NPs decoration. For monometallic NPs, the Au diffraction peaks are relatively sharp, whereas the Pd ones are broad or even absent. It is owing to the small size and resultant crystalline defects of the Pd NPs compared with the Au NPs [48, 49], which is consistent with the TEM results. Significantly, the Au diffraction peaks are slightly shifted toward larger 2*θ* side upon addition of Pd, and the Pd diffraction peaks gradually vanish upon the addition of Au. Both indicate the formation of PdAu bimetallic NPs rather than separate Au/Pd monometallic NPs, in good agreement with the above discussion based on the microscopic and electronic characterization results.

### Catalytic Behavior

**Fig. 6:**
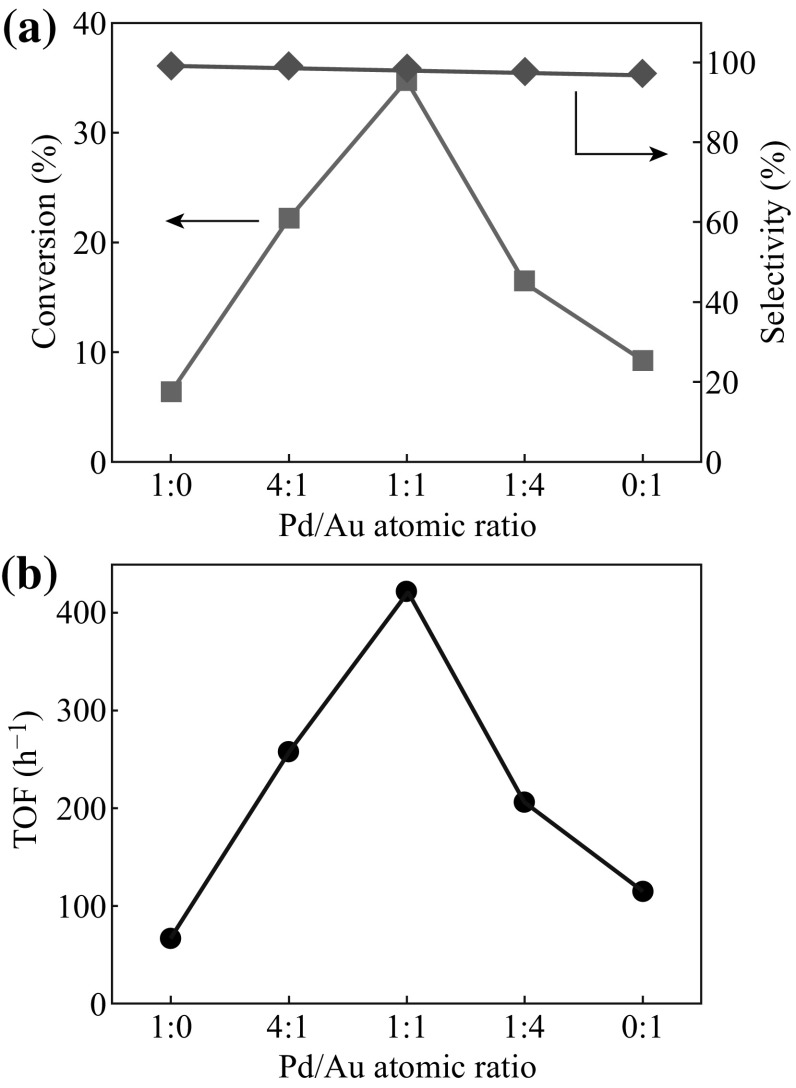
Dependences of catalytic activity for 1-phenylethanol oxidation on Pd/Au atomic ratio in TiO_2_-supported metal NPs: **a** conversion (*square*) and selectivity (*diamond*); **b** turnover frequency (*circle*). The catalytic reaction is carried out at 323 K and 1 atm using O_2_ as the oxidant

Utilizing the present RTIL-assisted sputtering method, PdAu bimetallic NPs on TiO_2_ with small size, bare surface, and controlled Pd/Au ratio are achieved. The catalytic activity for 1-phenylethanol oxidation of the metal–NPs–TiO_2_ hybrids is systematically studied, and the conversion and selectivity results are shown in Fig. 6a. Note that the catalytic reaction is realized using O_2_ as the oxidant at temperature as low as 50 °C and pressure as low as 1 atm. Furthermore, for a reliable comparison in catalytic activity, the total amount of sputtered metal is controlled to be the same for all the hybrids. Firstly, the Au monometallic NPs appear to be more active than the Pd monometallic NPs. Secondly, all the PdAu bimetallic NPs show higher activity compared with the monometallic counterparts. Thirdly, the conversion from 1-phenylethanol to acetophenone is strongly dependent on Pd/Au ratio, and it gets a maximum of about 35 % at 1:1 Pd/Au ratio. As a more catalytically relevant parameter, the highest TOF of ~421 h^−1^ is obtained at 1:1 Pd/Au ratio as well (Fig. 6b). Finally, the selectivity remains high irrespective of Pd/Au ratio.

The catalytic results suggest that the surface Au diluted by Pd is the active sites, and the synergistic effect can be understood in the following. On one hand, the size of the metal NPs is reduced with increasing Pd/Au ratio, which induces more surface area for the catalytic reaction. Furthermore, the dilution of Au by Pd generates more Au/Pd interface and isolated Au surface, which can promote the catalytic activity of Au (ensemble effect) [38, 50–52]. On the other hand, when Pd/Au ratio is high enough, the number of the Au active sites may decrease. In addition, owing to the charge redistribution effect in PdAu NPs, the *d*-hole depletion of Au is enhanced with increasing Pd/Au ratio. It may weaken the reactant adsorption on Au, which is adverse to the oxidation catalysis. As a consequence, the combined action of the above promotion and suppression effects shows the highest activity at 1:1 Pd/Au. The synergistic behavior indicates that Pd/Au ratio in PdAu NPs can be well tailored by the present means to optimize their catalytic performance.

## Conclusion

A green-chemistry compatible approach toward functional PdAu–NPs–TiO_2_ hybrids is demonstrated, and the hybrids show high catalytic activity for solvent-free synthesis of acetophenone from 1-phenylethanol. The catalytic reaction is realized under mild conditions, using O_2_ as the oxidant at the low temperature of 50 °C and low pressure of 1 atm. The approach for the catalyst preparation is straightforward and totally free of additives and byproducts. All the microscopic structure, electronic structure, and crystalline structure characterizations indicate the bimetallic formation of the TiO_2_-supported PdAu NPs due to atomic inter-diffusion. The catalytic activity of the PdAu NPs exhibits a strong dependence on Pd/Au ratio, which is tunable using the present RTIL-assisted sputtering method.
